# Supplementary material for: Conformational Insight on WT- and Mutated-EGFR Receptor Activation and Inhibition by Epigallocatechin-3-Gallate: Over a Rational Basis for the Design of Selective Non-Small-Cell Lung Anticancer Agents
Source: Int J Mol Sci. 2020 Mar 3;21(5):1721. doi: 10.3390/ijms21051721 (PMC7084708; doi:10.3390/ijms21051721)
Supplement: Supplementary file 1 [file ijms-21-01721-s001.pdf]

International Journal of *Molecular Sciences*

Supplementary Material

1. EGFRs structure modeling (SM)

The conformation of the small lacking loops between the crystallized domains of EGFR-TK receptor, and of the other fragments of EGFR has been well defined using a comparative modeling approach. On the contrary, a disordered structure corresponds to the small 16aa loop (Tyr975-Asp991) (yellow in the figure below), but this part is retrieved from the crystallographic structures 5cnn and 4rj4. Worth of notice, the sequence (Pro848-Pro877), is retrieved for the pdb template, since it is included as the final part of the TK-crystallized domains. This loop is directly involved in an important aa mutation (Leu858-Thr858), which confers to the EGFR drug resistance; thus, this portion associated with high degrees of freedom, must be extensively explored through a MD simulation approach. In addition, the structure of the missing aminoacids (Thr892-Tyr1197) and other flexible domains were also obtained.

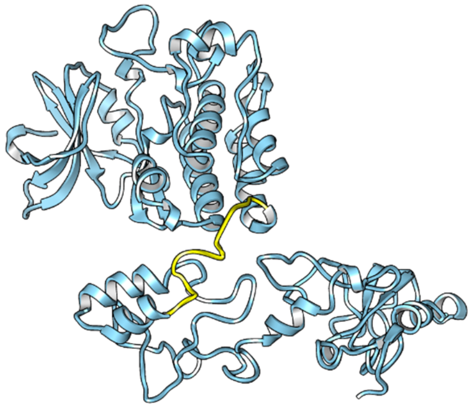

2. Post MD analyses

Specific studies aimed to better understand the movements of the dimerization domain of the three EGFR forms have been carried out, and the mean square displacement (MSD) of dimerization domain from the set of initial positions has been computed. This analysis provided us an easy way to determine the diffusion constant using the Einstein relation, which is a predictive relation about diffusive movements of particles subjected to a force field. It can be expressed in the form

$$\mathbf{D = \mu\, k_B\, T}$$

Where:

D is diffusive coefficient of matter

μ is mobility of particle, which is a ratio between speed limit and a force applied to it

k_B is the Boltzmann constant

T is the temperature (kelvin)

3. Comparison between 4HJO pdb structure and wild type EGFR model.

The generated EGFR wild type model was superimposed on tyrosin kinase domain reported in 4HJO pdb structure with root mean square deviation (RMSD) of 0.591 Å, showing a close homology (Figure S1A). The

geometry of entire cytoplasmic EGFR model was evaluated with Ramachandran plot calculation using RAMPAGE. Ramachandran plot revealed that 82 % of residues are in favored region, 14.4% are in additionally allowed region and 4.6% are in outlier region (Figure S1B). From Verify 3D analysis, it results 93.10% of the residues have averaged 3D-1D score >= 0.2.

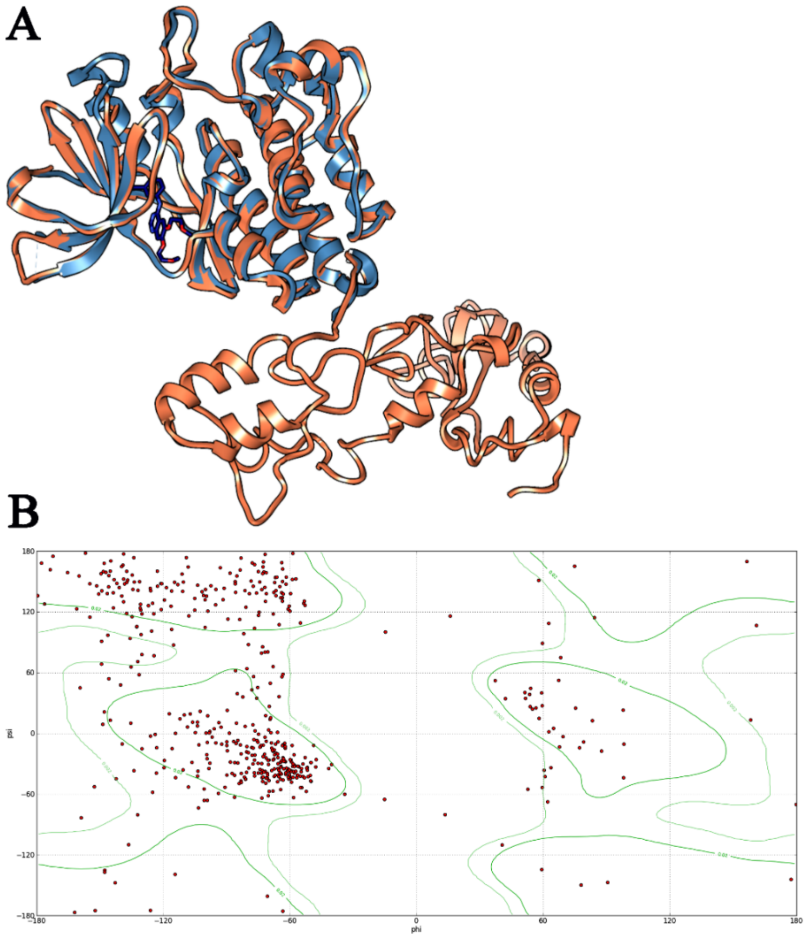

Figure S1. Superimposition of wild type EGFR model (orange) and 4HJO pdb template (blue); in dark blue is reported Erlotinib molecule in the ATP binding site (A). Ramachandran plots of wild type EGFR model.

4. RMSD results on wilt type, T790M/L858R EGFR cytoplasmic models

The root mean square deviation for the backbone atoms of wild type, T790M/L858R and ELREA deletion EGFR forms through the entire MD trajectory (RMSD vs Time) was calculated, and a stabilization was reached for all the EGFR forms at the end of the simulation time. In fact, the reached ΔRMSD values (figure 2) were very low, as the systems converged to 1 ± 0.05 nm (wild type model), 1.01 ± 0.04 nm (T790M/L858R model) and 0.87 ± 0.07 nm (ELREA Deletion model). In the first 20 ns of the simulation trajectories, all EGFR systems had RSMD values comparable between them, reaching a common steady state and indicating a high stabilization degree. This result is very important because it allowed us to validate our prediction method, because the RMSD values of around 1 nm for the entire protein structure 450 aa long (445 aa for ELREA model) are relative to stable systems.

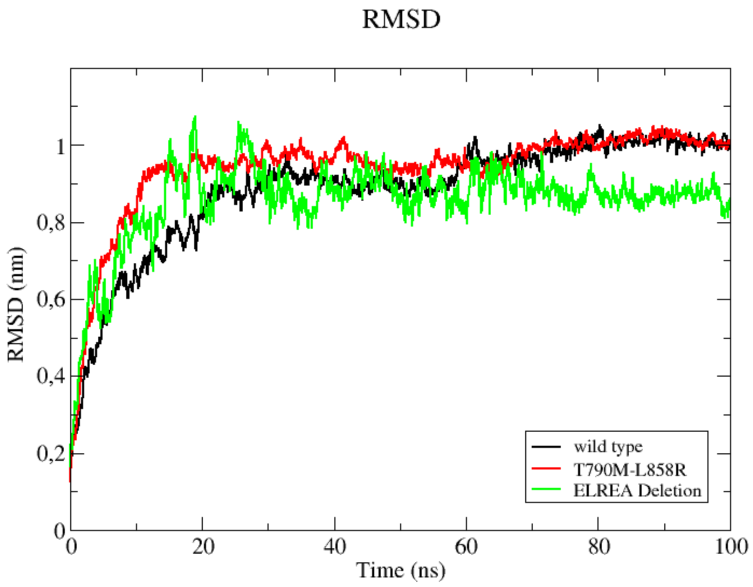

Figure S2. Root mean square deviation of EGFR cytoplasmic different forms

To better validate the goodness degree of prediction, 100 ns of MD simulation were repeated three times for EGFR receptors. Three EGFR structures were extrapolated from 20 ns, 50 ns and 70 ns along first MD simulation and then used as starting points for other 100 ns of MD simulations. Stabilization pathways of TK and D domains were investigated separately.

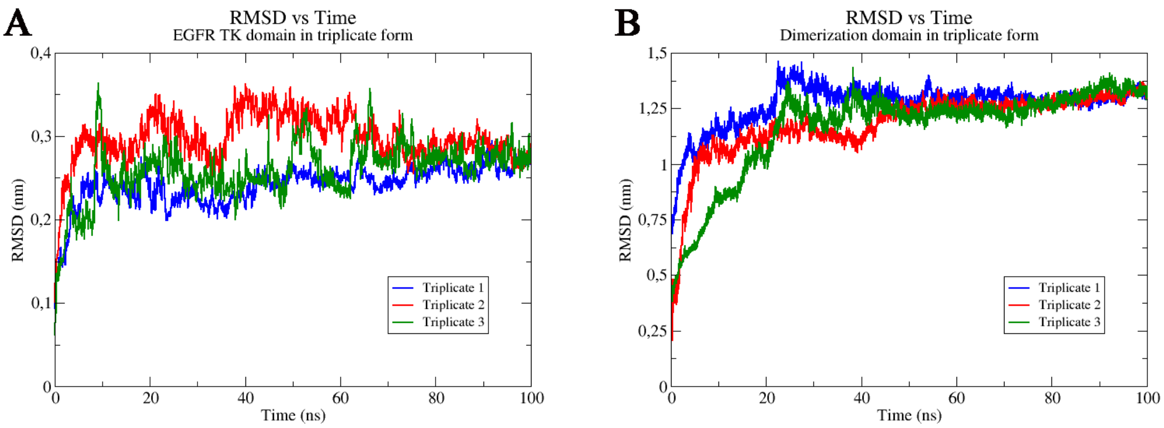

Figure S3. Root mean square deviation of TK (a) and D (b) domains of wt-EGFR for each of the triplicate MD runs. The triplicate 1,2 and 3 correspond to MD started from 20, 50 and 70 ns respectively form the first simulation.

The same values of RMSD were observed along simulation time, and TK domain confirmed to be an ordered rigid domain, with RMSD values lower than 0.3 nm for all triplicates (figure S3A). The D domain showed a more flexibility, with RMSD values higher than 1.25 nm (figure S3B). However, the same values were found for triplicates, confirming the good degree of prediction.

5. Combined molecular docking/Molecular dynamics approach

The EGFR cytoplasmic forms obtained after MD stabilization (corresponding to the steady state) were used for molecular docking, at the aim to create the EGFR inactive forms in complexes with ligands considered.

5.1. wild type EGFR complexes

Analyzing the Michaelis complexes obtained after 20 ns of MD trajectories, some important differences have been found comparing Erl and EGCG dynamical binding mode. In the last 5 ns of simulations, Erl showed a RMSD value of 0.13 ± 0.05 Å, while EGCG showed a value of 0.12 ± 0.01 Å. (Figure 3A) The substantial difference is related to the estimated error, which is 5 times higher than that seen for system with EGCG. This means a significantly higher fluctuation for Erl than that observed for EGCG in the ATP binding site. From these data, EGCG showed a more efficient binding mode respect to Erl, as confirmed by RMSD value of total backbone of EGFR in association with two ligands studied (figure 3B). The EGFR-Erl complex showed values of 1.5 ± 0.1 nm in the last 5 ns of simulation, while the EGFR-EGCG system highlighted a 0.90 ± 0.08 nm RMSD value. EGFR-EGCG complex showed a major stability respect to that obtained with Erl, due to important hydrophobic and hydrophilic stable interactions with different aminoacids.

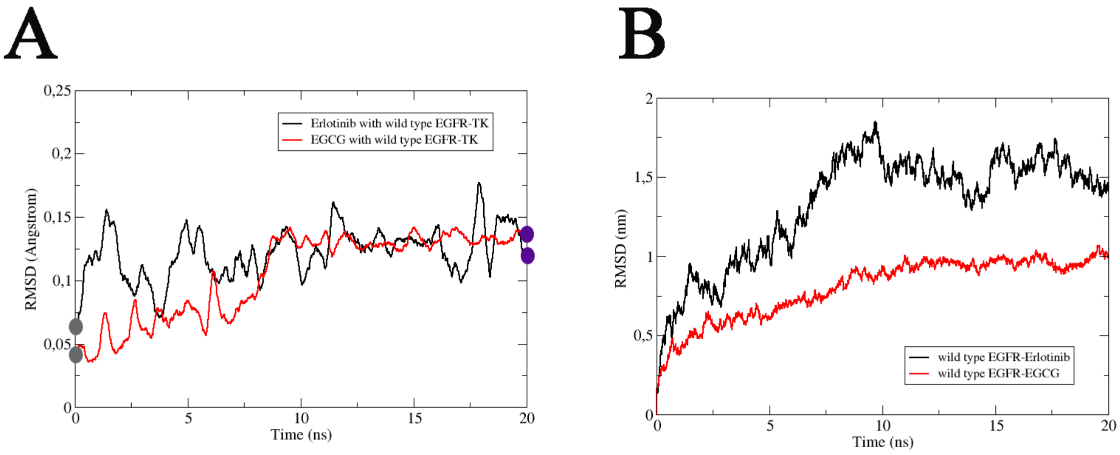

Figure S4. The root mean square deviations (RMSD) of ligands (A) and wild type EGFR backbone in complex with ligands (B) have been highlighted.

5.2. ELREA EGFR complexes

Analysing the RMSD values of ligands in association with ELREA EGFR structure, Erl adopted a new stable conformation after 10 ns of MD simulation (figure S4A), reached an RMSD value of 0.13 ± 0.01 Å. Different situation was found for EGCG binding mode; the catechin molecule adopted the same conformation analyzing MD trajectories, but no particular stability was found, because the RMSD value reached 0.18 ± 0.04 Å, with a higher extimated error, and then an higher fluctuation degree in the ATP binding siteThis different behavior of two ligands influenced the stabilization pathway of the entire ELREA deletion EGFR (figure 4B). The complex with Erl reached stability after 10 ns of MD simulation, in relation to the new stable conformation adopted for the ligand, with an RMSD value of 0.34 ± 0.01 nm. Similarly, EGCG determined different stabilization pathway for receptor, with RMSD value for the last 5 ns was 0.72 ± 0.06 nm.

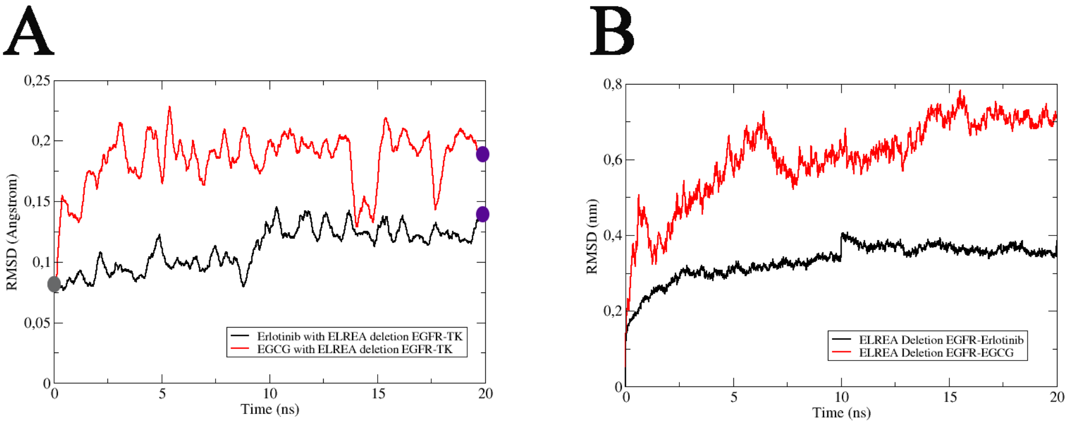

Figure S5. The root mean square deviations (RMSD) of ligands (a) and ELREA deletion EGFR backbone in complex with ligands (b) have been highlighted.

5.3. T790M/L858R EGFR complexes

Analysing the RMSD data obtained from MD simulation, two different stabilization pathways have been found for Erl and EGCG (figure S5A). The first compound showed a completely different conformation adopted after 8 ns of MD simulation, reaching an RMSD value of 0.22 ± 0.03 Å. The EGCG molecule showed the same RMSD value for all 20 ns of simulation, indicating no variations in the binding mode. EGCG appeared more able than Erl to efficacy interact with resistant EGFR form, but low hydrophobic contribution in the binding energy was observed. No peculiar results have been obtained analysing the RMSD value for the backbone of EGFR in complex with the two ligands (figure 5B). Both of systems reached average RMSD values of about 0.8 nm at the end of simulation but, with important deviations during the last 10 ns, showing no efficent stabilization pathway. These data and the low hydrophobic contribution found for both EGFR-Erl and EGFR-EGCG complexes indicated that neither of the two ligands studied seemed to be able to efficacy inhibit the T790M/L858R resistant form of the EGFR-TK domain.

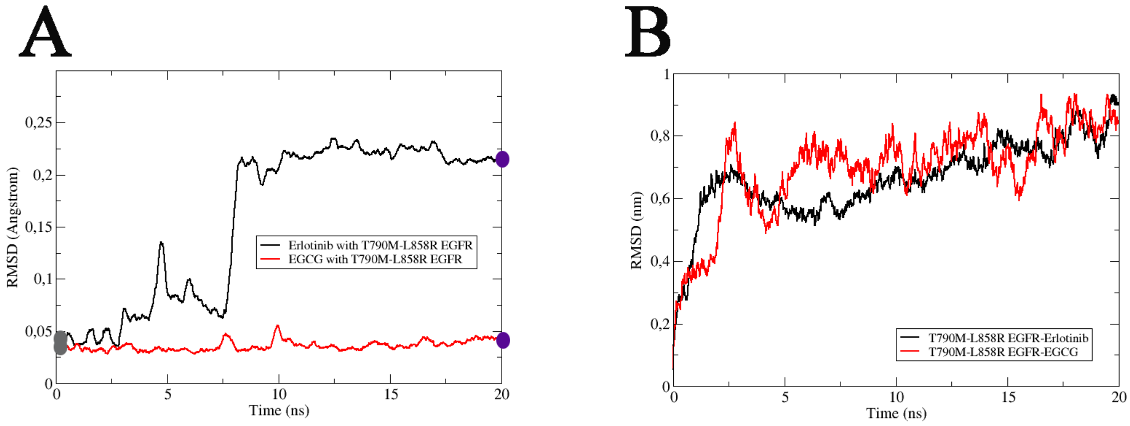

Figure S6. The root mean square deviations (RMSD) of ligands (A) and T790M/L858R EGFR backbone in complex with ligands (B) have been highlighted.
